# Mapping evidence on ovarian, endometrial, vaginal, and vulva cancer research in Africa: a scoping review protocol

**DOI:** 10.1186/s13643-021-01654-0

**Published:** 2021-04-14

**Authors:** Sebastian Yidana Ninimiya, Monica Ansu-Mensah, Vitalis Bawontuo, Desmond Kuupiel

**Affiliations:** 1 Faculty of Health and Allied Sciences, Catholic University College of Ghana, Fiapre, Sunyani, Ghana; 2 St. Theresa’s Hospital, Nandom, Upper West Region Ghana; 3 Sunyani Technical University, Sunyani, Bono Region Ghana; 4 Centre for Evidence-based Health Care, Division of Epidemiology and Biostatistics, Department of Global Health, Faculty of Medicine and Health Sciences, Stellenbosch University, Cape Town, 7530 South Africa; 5 Research for Sustainable Development (r4ds) consult, Sunyani, Ghana

**Keywords:** Gynecological cancer, Ovarian, Endometrial, Rare gynecological neoplasms, Screening techniques, Epidemiological burden, Risk factors, Africa

## Abstract

**Background:**

Globally, cancer is generally recognized as a developmental threat yet most countries in Africa lack capacity to diagnose cancer especially gynecological cancers resulting in late detection and poor outcomes. However, most studies on gynecological cancers in Africa tend to focus on cervical cancer compared to the other gynecological cancers. Therefore, this scoping review will aim to describe the existing literature on the epidemiological burden of ovarian, endometrial, vaginal, and vulva cancers, their risk factors, and potential screening methods/techniques in Africa to identify priority research gaps for further research to inform health policy decisions.

**Methods:**

The framework promulgated by Arksey and O’Malley and improved by Levac et al. will be used as a guide for this scoping review. A comprehensive search for relevant published studies in PubMed, CINAHL, SCOPUS, Google Scholar, and ScienceDirect with no date limitation to the last search date. The database search strategy will include keywords, Boolean operators, and medical subject heading terms. We will additionally consult the WHO/IARC website, IHME/Global Burden of Disease Study. A snowball approach will also be used to search the reference list of all included studies to obtain relevant papers for possible inclusion in this review. We will include articles that involve African countries, focused on ovarian, endometrial, vaginal, and vulva cancers, their risk factors, and potential screening methods/techniques in any language. We will exclude studies on cervical cancer and other cancers as well as review articles. The abstracts and full-text selection will be conducted by two independent reviewers using this review’s eligibility criteria as a guide. All the review selection tools, and the data extraction form will be pilot tested for accuracy and consistency. The data will be organized into thematic areas, summarized and the results communicated narratively.

**Discussion:**

It is anticipated that this review will reveal important literature gaps to guide future research to inform health policy decisions about ovarian, endometrial, and rare gynecological neoplasms in Africa. This review’s findings will be disseminated via peer review journals, conferences, and other social media such Twitter and LinkedIn.

## Background

Cancer is generally recognized as a threat to global development. In 2017, the global incidence and deaths due to cancer were estimated to be approximately 24.5 million cases and 9.6 million deaths respectively [1]. Cancer is part of the emerging non-communicable diseases in Africa with an ever-increasing burden on individuals, communities, and healthcare systems. A higher number of people die from cancers compared to acquired immunodeficiency syndrome, tuberculosis, and malaria combined [2]. Cancer (including gynecological cancers) hitherto was thought to be a disease of the affluent particularly those in high-income countries, but there are indications that the burden of cancer is rising in low-and-middle-income countries (LMICs) despite facing diagnostic challenges [2]. In 2018, the global cancer incidence, mortality, and prevalence, number (GLOBOCAN) report showed a steady rise in the occurrence of gynecological cancers in LMICs [3].

Gynecological cancers include cervical cancer, endometrial cancer, ovarian and fallopian tube cancers, cancer of the vagina, and cancer of the vulva. Primary vaginal cancer is relatively rare but is associated with HIV infection which is highly prevalent in Africa [4]. Most vaginal cancers are secondary, but chronic human papillomavirus (HPV) infections which are rife in Africa results in primary vaginal cancer [4, 5]. Vulva cancer also relatively rare has a causal relationship with chronic oncogenic HPV infections and preventive strategies aimed at cervical cancer has benefits for vulva cancer [6, 7]. Endometrial cancer is the third commonest gynecological cancer in LMICs [2]. The risk factors of these cancers may include a sedentary lifestyle, obesity, unopposed estrogen, infertility, and decreased fertility rates [8, 9]. With a growing westernization in the lifestyle of Africans, these risk factors are increasingly becoming important in the continent. Ovarian cancer with about 18.8% representation of all gynecological cancers in LMICs [3] is characterized by a high case fatality rate because it is largely asymptomatic until the late stages of the disease [10]. Fallopian tube cancers have similar characteristics with ovarian cancers and are classified by the International Federation of Gynaecology and Obstetrics together with ovarian cancers [10].

A prior review indicated an increasing burden of gynecological cancers in LMICs due to a rise in cervical cancer incidence [11]. In 2013, Iyoke and Ugwu conducted a review and reported that 60% of gynecological cancers in these countries which are mostly found in Africa are due to cervical cancer [11]. The highest incidence and mortality of cervical cancers occur in African countries [4] mostly due to the absence of effective national preventive strategies in these countries [11]. Together with vaginal cancer and cancer of the vulva, cervical cancer is highly preventable with vaccination against the highly oncogenic HPV species [4, 6, 7, 12]. However, poor diagnostic abilities in most African countries coupled with general poor health-seeking behavior is likely to hamper early detection and desired treatment outcomes of cancer including most gynecological cancers. Although several gynecological cancers exist, much attention has been given to cervical cancer compared to the others particularly in Africa due to known reasons including diagnostic challenges. Nonetheless, research on the other gynecological neoplasms such as ovarian, endometrial, and other rare gynecological neoplasms is essential. Several previous reviews have been published in the past relating to gynecological cancer screening [11, 13–15] but to date, the range of research and knowledge gaps on ovarian, endometrial, vaginal, and vulva cancers in Africa is not known. Therefore, we propose to conduct a scoping review to describe evidence about gynecological cancers in Africa focusing on the epidemiological burden of ovarian, endometrial, vaginal, and vulva cancers, their risk factors, and potential screening methods/techniques. We anticipate that this review will reveal important literature gaps to inform future research to inform health policy decisions about ovarian, endometrial, and rare gynecological neoplasms in Africa.

## Methods

### Overview of the study

Scoping reviews aim to map rapidly the key concepts underpinning a research area and the main sources and types of evidence available. Scoping reviews also involve the synthesis and analysis of a wide range of research and non-research material to provide greater conceptual clarity about a specific topic or field of evidence [16–18]. Scoping reviews aim to provide a map of what evidence has been produced from disparate or heterogeneous sources as opposed to seeking only the best evidence to answer a particular question related to policy or practice [18]. This review will be based on the 2005 Arksey and O’Malley framework as reviewed and enhanced by Levac and colleagues in 2010 [19–21]. This protocol stipulates 5 mandatory and 1 optional steps as follows: identification of research question; identification of relevant studies; study selection; charting of data, collating, summarizing and reporting the results; and the optional step of consultation [20–22]. This protocol is reported according to the Preferred Reporting Items for Systematic Reviews and Meta-Analysis Protocol (PRISMA-P) statement [23]. However, the resulting paper will be reported per the PRISMA extension for Scoping Reviews checklist [21].

### Identifying the research question

Our overall scoping review question will be: What evidence exists on ovarian, endometrial, vaginal, and vulva cancer research in Africa? The population, concept, and context (PCC) mnemonic (Table 1) was used to determine the eligibility of the review question. The secondary research questions include:

- What evidence exists on the epidemiological burden (morbidity, incidence, prevalence, and mortality) of ovarian, endometrial, vaginal, and vulva cancers in Africa?
- What evidence exists on the risk factors of ovarian, endometrial, vaginal, and vulva cancers in Africa?
- What evidence exists on potential screening techniques/methods/recommendations for ovarian, endometrial, vaginal, and vulva cancers in Africa?

**Table 1:** PCC defining the eligibility of the main scoping review question

P-Population	Women
C-Concept	Gynecological neoplasms: This will include ovarian, endometrial, vaginal, and vulva cancers
C-Context	The epidemiological burden (incidence, morbidity, prevalence, and mortality); risks factors; and potential screening methods/techniques in African countries

### Information sources and search strategy

A comprehensive search for relevant published articles on gynecological cancers in Africa will be conducted in the following databases: PubMed/MEDLINE, CINAHL, Web of science, SCOPUS, Google Scholar, and ScienceDirect without date limitation to the last search date. The search will include the use of a combination of relevant keywords and index terms with appropriate Boolean operators (AND/OR). Medical Subject Headings (MeSH) terms or subject heading in all fields will be included and syntax modified appropriately for each database if necessary, to enable identification of all relevant studies. Study design and publication language limitations will be removed during the search. An electronic pilot search strategy for this review is illustrated in Table 2. Nonetheless, the authors will consult an experienced librarian to improve the search strategy if needed. We will also consult the websites of the WHO/International Agency for Research on Cancer, Institute for Health Metrics and Evaluation/Global Burden of Disease Study. A snowball approach will further be used to search the reference list of all included articles to obtain relevant articles for possible inclusion in this review. A detailed searched record will be documented as follows: date of search, search engine used, keywords used in the search, the number of retrieved publications, and the number of eligible studies found. The principal investigator (SYN) who is an expert obstetrician and gynecologist will conduct the search in the electronic databases. Mendeley Desktop reference manager will be used to compile all the articles deemed relevant for this review.

**Table 2:** Pilot search for the review conducted on PubMed

Date	Database	Keywords	Search results
18/09/2020	PubMed	(((((“Gynecological cancer”[MeSH Terms]) OR “cervical cancer”[MeSH Terms]) OR “endometrial cancer”[MeSH Terms]) OR “ovarian cancer”[MeSH Terms]) OR “fallopian tube cancers”[MeSH Terms]) OR “cancer of the vagina”[MeSH Terms]) OR “cancer of the vulva”[MeSH Terms]) OR “screening service”[MeSH Terms]) OR “screening”[MeSH Terms]) AND africa) OR “angola”[MeSH Terms] OR “angola”[All Fields]) OR (“benin”[MeSH Terms] OR “benin”[All Fields])) OR (“botswana”[MeSH Terms] OR “botswana”[All Fields])) OR (“burkina faso”[MeSH Terms] OR (“burkina”[All Fields] AND “faso”[All Fields]) OR “burkina faso”[All Fields])) OR (“burundi”[MeSH Terms] OR “burundi”[All Fields])) OR (“cameroon”[MeSH Terms] OR “cameroon”[All Fields])) OR (“cabo verde”[MeSH Terms] OR (“cabo”[All Fields] AND “verde”[All Fields]) OR “cabo verde”[All Fields] OR (“cape”[All Fields] AND “verde”[All Fields]) OR “cape verde”[All Fields])) OR (“central african republic”[MeSH Terms] OR (“central”[All Fields] AND “african”[All Fields] AND “republic”[All Fields]) OR “central african republic”[All Fields])) OR (“chad”[MeSH Terms] OR “chad”[All Fields])) OR (“comoros”[MeSH Terms] OR “comoros”[All Fields])) AND (“congo”[MeSH Terms] OR “congo”[All Fields])) OR (“cote d'ivoire”[MeSH Terms] OR (“cote”[All Fields] AND “d'ivoire”[All Fields]) OR “cote d'ivoire”[All Fields])) OR (“djibouti”[MeSH Terms] OR “djibouti”[All Fields])) OR (“equatorial guinea”[MeSH Terms] OR (“equatorial”[All Fields] AND “guinea”[All Fields]) OR “equatorial guinea”[All Fields])) OR (“eritrea”[MeSH Terms] OR “eritrea”[All Fields])) OR (“ethiopia”[MeSH Terms] OR “ethiopia”[All Fields])) OR (“gabon”[MeSH Terms] OR “gabon”[All Fields])) OR (“gambia”[MeSH Terms] OR “gambia”[All Fields] OR “the gambia”[All Fields])) OR (“ghana”[MeSH Terms] OR “ghana”[All Fields])) OR (“guinea”[MeSH Terms] OR “guinea”[All Fields])) OR (“guinea-bissau”[MeSH Terms] OR “guinea-bissau”[All Fields] OR (“guinea”[All Fields] AND “bissau”[All Fields]) OR “guinea bissau”[All Fields])) OR (“kenya”[MeSH Terms] OR “kenya”[All Fields])) OR (“lesotho”[MeSH Terms] OR “lesotho”[All Fields])) OR (“liberia”[MeSH Terms] OR “liberia”[All Fields])) OR (“madagascar”[MeSH Terms] OR “madagascar”[All Fields])) OR (“malawi”[MeSH Terms] OR “malawi”[All Fields])) OR (“mali”[MeSH Terms] OR “mali”[All Fields])) OR (“mauritania”[MeSH Terms] OR “mauritania”[All Fields])) OR (“mauritius”[MeSH Terms] OR “mauritius”[All Fields])) OR (“mozambique”[MeSH Terms] OR “mozambique”[All Fields])) OR (“namibia”[MeSH Terms] OR “namibia”[All Fields])) OR (“niger”[MeSH Terms] OR “niger”[All Fields])) OR (“nigeria”[MeSH Terms] OR “nigeria”[All Fields])) OR (“rwanda”[MeSH Terms] OR “rwanda”[All Fields])) OR (“sao tome and principe”[MeSH Terms] OR (“sao”[All Fields] AND “tome”[All Fields] AND “principe”[All Fields]) OR “sao tome and principe”[All Fields])) OR (“senegal”[MeSH Terms] OR “senegal”[All Fields])) OR (“seychelles”[MeSH Terms] OR “seychelles”[All Fields])) OR (“sierra leone”[MeSH Terms] OR (“sierra”[All Fields] AND “leone”[All Fields]) OR “sierra leone”[All Fields])) OR (“somalia”[MeSH Terms] OR “somalia”[All Fields])) OR (“south africa”[MeSH Terms] OR (“south”[All Fields] AND “africa”[All Fields]) OR “south africa”[All Fields])) OR (“sudan”[MeSH Terms] OR “sudan”[All Fields])) OR (“eswatini”[MeSH Terms] OR “eswatini”[All Fields] OR “swaziland”[All Fields])) OR (“tanzania”[MeSH Terms] OR “tanzania”[All Fields])) OR (“togo”[MeSH Terms] OR “togo”[All Fields])) OR (“uganda”[MeSH Terms] OR “uganda”[All Fields])) OR (“zambia”[MeSH Terms] OR “zambia”[All Fields])) AND (“zimbabwe”[MeSH Terms] OR “zimbabwe”[All Fields]) AND (“2013/01/01”[PubDate] : “2020/12/31”[PubDate])	15538

### Study selection and eligibility criteria

#### Study selection

**Fig. 1:**
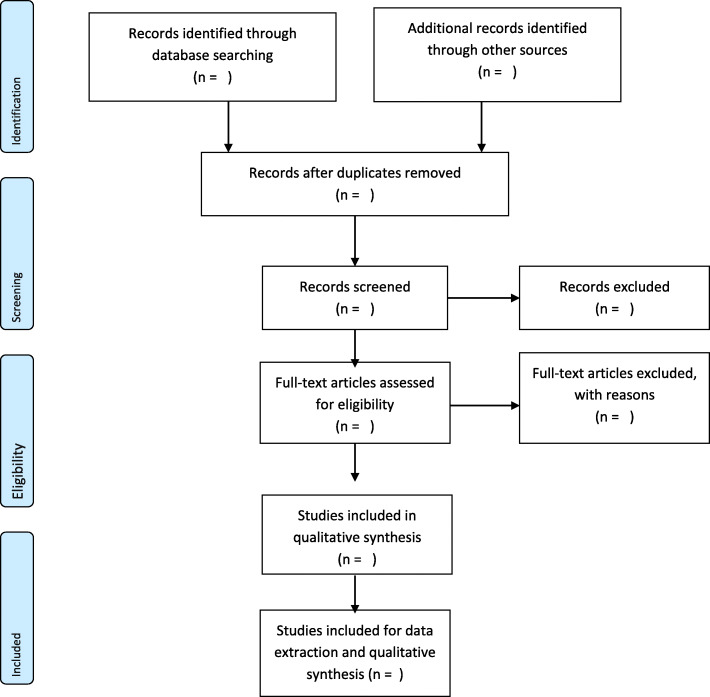
PRISMA 2009 flow diagram

The principal investigator (SYN) will conduct an extensive title screening guided by this review’s eligibility criteria and all eligible studies imported to a folder created in Mendeley reference manager for this scoping review study. The library created will be cleaned by identifying and removing duplicate articles before the abstract screening phase. Subsequently, the cleaned library will be shared among the review team. SYN and MAM will independently screen the abstract and full text using the eligibility criteria for the review. Disagreements between SYN and MAM responses shall be resolved by dialog and consensus-building. DK will serve as an arbiter at the full-text screening stage if any disagreement arises between SYN and MAM. In the case where a full-text article cannot be retrieved or is not accessible from the online databases, assistance will be sought from the Catholic University College library or the Stellenbosch University to obtain it for screening. We will also consider writing to the authors to request any full-text article not accessible freely online if needed. Finally, this review will adopt the PRISMA flow chart [22, 23] (Fig. 1) to account for the articles at each stage of the screening.

#### Eligibility criteria of this review

##### Inclusion criteria

This will include the following:

- Studies that involved African countries
- Studies that include human participants (women)
- Studies that include ovarian, endometrial, vaginal, and vulva cancers
- Studies reporting on the epidemiological burden (morbidity, incidence, prevalence, and mortality) of ovarian, endometrial, vaginal, and vulva cancers
- Studies that reported findings on the risk factors of ovarian, endometrial, vaginal, and vulva cancers
- Primary study designs (quantitative, qualitative, and mixed methods studies)

##### Exclusion criteria

This will include the following:

- Studies that focused on other gynecological cancers such as cervical cancer
- Studies that focused on other types of cancer such as breast cancer
- Studies focus on the natural science of ovarian, endometrial, vaginal, and vulva cancers
- Studies focusing on the diagnostic and/management/treatment of ovarian, endometrial, vaginal, and vulva cancers
- Studies focusing on the economic burden of ovarian, endometrial, vaginal, and vulva cancers
- Gray literature, conference papers, opinions, editorials, theses, dissertations, and unpublished studies
- Articles without full text

### Charting the data

We will extract all the germane findings from the included articles aimed at answering the main scoping review question. Two reviewers (SYN and MAM) will independently pilot the data extraction form using 10 percent of the included studies to ensure consistency and uniformity. Appropriate adjustments will consequently be made for final use. The data extraction form will be updated regularly until all relevant and applicable information has been extracted. The data extraction form will include the following:

- Author and publication year
- Study title
- Aim/objective of the study
- Geographical location of the study (country)
- Type of study design
- Study setting (such as facility-based, community-based, others)
- Number of study participants
- Mean age/range of study population (women)
- Targeted gynecological cancer
- Burden (incidence, prevalence, morbidity, mortality)
- Reported risk factors per the type of gynecological cancer investigated
- Potential screening techniques/methods/recommendations
- Relevant recommendations/conclusions

### Collating, summarizing, and reporting the results

Thematic content analysis will be employed to describe the themes that are related to this review’s objective. The thematic analysis would ensure the identification of all the themes essential to address the research question. The sub-themes will be collated and structured around the following main themes: the epidemiological burden (incidence, morbidity, prevalence, and mortality) of ovarian, endometrial, vaginal, and vulva cancers in Africa; and the reported risk factors of ovarian, endometrial, vaginal, and vulva cancers in Africa; and potential screening methods/techniques for ovarian, endometrial, vaginal, and vulva cancers in Africa. This review will also analyze other relevant emerging themes. Subsequently, we will summarize the findings and a narrative account for each theme or outcome reported. A map will be used to show the geographical locations of the included studies. Graphs and tables will also be used to present the study characteristics such as the study designs, and others where possible. If feasible, a follow-up meta-analysis using quantitative data from this review will be considered due to the qualitative nature of scoping review studies.

## Discussion

This scoping review study aims to describe the existing literature on the epidemiological burden of ovarian, endometrial, vaginal, and vulva cancers, their risk factors, and potential screening methods/techniques in Africa. Cancer is a global health problem and remains a threat to development but many countries in low-and-middle-income countries (LMICs) lack capacity to diagnose most cancers including gynecological cancers which often result in late detection and poor outcomes. Research relating to gynecological cancers in many LMICs tends to focus more on cervical cancer although several other gynecological cancers exist. Knowledge of the range of evidence or research activity on the other gynecological cancers aside from cervical cancer is essential to help identify priority research gaps for future research to facilitate health policy decisions, hence, the need for this review. Due to lack of external funding, this review will be limited to African countries only though the inclusion of other LMICs would have been great. Similarly, we will not cover research on diagnostic and/management/treatment of ovarian, endometrial, vaginal, and vulva cancers as well as other rare gynecological cancers. The evidence obtained from this review will be disseminated via publication on several platforms including peer-reviewed journals, conferences, and interactions with potential knowledge users.

## Data Availability

We have duly cited all studies and data is presented in the form of references.

## Abbreviations

LMICs: Low-and-middle-income countries
HPV: Human papilloma virus
HIV: Human immunodeficiency virus
MMAT: Mixed methods appraisal tool
